# A systematic practice review: Providing palliative care for people with Parkinson’s disease and their caregivers

**DOI:** 10.1177/02692163231214408

**Published:** 2023-12-06

**Authors:** Michela Garon, Christiane Weck, Kristina Rosqvist, Per Odin, Anette Schrag, Ülle Krikmann, David J Pedrosa, Angelo Antonini, Stefan Lorenzl, Sandra Martins Pereira, Piret Paal

**Affiliations:** 1 Parkinson and Movement Disorders Unit, Study Center for Neurodegeneration (CESNE), Department of Neuroscience, University of Padua, Via Giustiniani 2, 35128, Padua, Italy; 2 Padua Neuroscience Center (PNC), University of Padua, 35131, Padua, Italy; 3 Parkinson’s Disease and Movement Disorders Unit, Center for Rare Neurological Diseases (ERN-RND), Department of Neurosciences, University of Padova, Padova, Italy; 4 Institute of Palliative Care, Paracelsus Medical University, Salzburg, Austria; 5 Division of Neurology, Department of Clinical Sciences Lund, Lund University, Lund, Sweden; 6 Department of Neurology, Rehabilitation Medicine, Memory and Geriatrics, Skane University Hospital, Lund, Sweden; 7 Department of Clinical Neurosciences, UCL Queen Square Institute of Neurology, University College London, London, UK; 8 Department of Neurology and Neurosurgery, Institute of Clinical Medicine, University of Tartu, Tartu, Estonia; 9 Philipps University Marburg, Marburg, Germany; 10 Center for Mind, Brain and Behavior, Marburg, German; 11 Ethics and Sustainability Research Area: Palliative Care Research, CEGE: Research Centre in Management and Economics, Católica Porto Business School, Universidade Católica Portuguesa, Porto, Portugal

**Keywords:** Parkinson’s disease, clinical guideline, palliative care, caregivers, quality of life

## Abstract

**Background:**

People with Parkinson’s disease has significant and increasing physical, psychosocial and spiritual needs, as well as problems with coordination and continuity of care. Despite the benefits that palliative care could offer, there is no consensus on how it should be delivered.

**Aim:**

The aim of this study is to provide a pragmatic overview of the evidence to make clinical recommendations to improve palliative care for people with Parkinson’s disease and their caregivers.

**Design:**

A systematic review method was adopted to determine the strength of evidence, supported by feedback from an expert panel, to generate the ‘do’, ‘do not do’ and ‘do not know’ recommendations for palliative care.

**Data sources:**

Searches were conducted via OVID to access CINAHL, MEDLINE, EMBASE and the Cochrane Library from 01/01/2006 to 31/05/2021. An additional search was conducted in December 2022. The search was limited to articles that included empirical studies of approaches to enabling palliative care.

**Results:**

A total of 62 studies met inclusion criteria. There is evidence that education about palliative care and movement disorders is essential. palliative care should be multi-disciplinary, individualised and coordinated. Proactive involvement and support of caregivers throughout the illness is recommended. Limited data provide referral indicators for palliative care integration. Discussions about advance care planning should be held early.

**Conclusions:**

Consideration of palliative care integration based on symptom burden and personal preferences, coordination and continuity of care are needed to maintain the quality of life of people with Parkinson’s disease and their caregivers.

**What is already known about the topic?**

- People with Parkinson’s disease experience serious health related suffering and have therefore right to palliative care
- The number of consultations by specialist palliative care for people with Parkinson’s disease is low
- The transition to palliative care is initiated at a late stage
- Informal caregivers play a crucial role in providing optimal patient care

**What this paper adds?**

- Practical recommendations to enable better palliative care for people with Parkinson’s disease
- Highlights challenges related to the care pathway, gender differences, ethnicity and symptoms in the pre-diagnostic phase
- With regard to healthcare providers and care coordination, there is a need for clear and timely information on available health and social care resources and on the roles and responsibilities of the different stakeholders involved in the care process
- Lists Parkinson’s-specific aspects that should be included in the Advanced Care Plan
- Indicates the need for support from informal caregivers if patients wish to remain at home despite their deteriorating health status
- Sets out triggers for referral to a specialist palliative care unit

**Implications for practice, theory and policy**

- Advanced Care Planning in people with Parkinson’s disease should determine how to optimise care in the final years of the disease. This is not usually a traditional palliative approach, but rather other types of advanced disease management that focus on non-motor symptoms and quality of life.
- Informal caregivers are overlooked as key players in providing optimal care for people with Parkinson’s disease. Therefore, caregivers’ experiences, resulting needs and support planning need to be regularly assessed.
- Support for informal caregivers in national policy to maintain their motivation to care should include the provision of care breaks, such as respite services.

## Introduction

Palliative care is often misunderstood and used interchangeably with end-of-life care, hospice care or perceptions of death and dying.^1^ However, the World Health Organization (WHO) definition clarifies that palliative care is a comprehensive approach to patient care focussed on improving the quality of life for patients and their families who are facing untreatable illnesses through the relief of physical, psychosocial and spiritual suffering.^2^ The relationship between the patient, carer and a multidisciplinary health care model is at the heart of palliative care, where collaboration and effective communication are essential for its success.^3^ Despite the presence of a growing body of literature supporting the adoption and efficacy of this approach to neurological illnesses, for example Parkinson’s Disease, it remains mostly not accessible.^4^ In this context, neuropalliative care represents a growing medical sub-specialty dedicated to advancing educational, research, clinical and advocacy initiatives with the goal of enhancing palliative care for individuals with neurological conditions.^4^ Neuropalliative care is focussed on identifying and exploring the opportunities to improve the quality of care, offering clear and sensitive communication, complex symptom management, spiritual and psychological care for patients and caregivers, advance care planning and care at the end of life.^3–5^ In the field of neuropalliative care, geographical, cultural, socio-economic, gender and ethnic inequalities have been identified in access to palliative care.^6,7^

People with Parkinson’s Disease have limited access to palliative care services, as the available services lack of specific Movement Disorder’s expertise and have primarily an oncology focus.^8^ Moreover, several challenges are posed by the great variability of the disease: its course and development, the difficulties in establishing a prognosis and in identifying the end stage of the disease.^9^ In fact, they are rarely referred to hospices, often hospitalised at the end of life without receiving the benefits of palliative care and frequently pass away in hospital settings.^1,10^ The variability and uniqueness of symptoms in Parkinson’s Disease significantly impact the quality of life for both patients and caregivers, starting from the early stages of the disease.^11^ This underscores the need for specialised expertise in the palliative approach.

## Aims

The aim of this systematic practice review is to provide healthcare professionals with a pragmatic overview of approaches and recommendations to provide better palliative care for people with Parkinson’s disease.

## Methods

This practice review was designed to provide an overview of the current evidence on providing palliative care for people with Parkinson’s disease and their caregivers, with a supporting evidence-based set of recommendations. This practice review was guided by an integrative systematic review, a type of systematic review that allows for the inclusion of diverse methodologies, for example, experimental and non-experimental research.^12,13^ The Preferred Reporting Items for Systematic Reviews and Meta-Analysis (PRISMA) was used as a reporting guideline,^14^ which we adapted for the purposes of this practice review. The protocol of this systematic integrative review was registered in PROSPERO: CRD42021254848.

### Search strategy

An online search was conducted via OVID to access CINAHL, Cochrane, EMBASE and MEDLINE, from 01/01/2006 to 31/05/2021. This timeframe was chosen since, in 2006, the World Health Organization published a policy document highlighting the challenges and burden of neurological conditions for public health.^15^ To update the initial search an additional search using the same search terms was made in December 2022.

The following core terms and searches are systematised in Box 1. Key search terms were used whenever possible in all databases. The search strings were adapted as necessary for each database.

**Box 1.** Core search terms and searches.

(‘parkinson disease’[MeSH Terms] OR (‘parkinson’[All Fields] AND ‘disease’[All Fields]) OR ‘parkinson disease’[All Fields] OR ‘parkinsons’[All Fields] OR ‘parkinson’[All Fields] OR ‘parkinson s’[All Fields] OR ‘parkinsonian disorders’[MeSH Terms] OR (‘parkinsonian’[All Fields] AND ‘disorders’[All Fields]) OR ‘parkinsonian disorders’[All Fields] OR ‘parkinsonism’[All Fields] OR ‘parkinsonisms’[All Fields] OR ‘parkinsons s’[All Fields]) AND (‘palliative care’[MeSH Terms] OR (‘palliative’[All Fields] AND ‘care’[All Fields]) OR ‘palliative care’[All Fields]).

The PICO (Population, Intervention, Context, Outcome) mnemonic (see Box 2) was applied to refine the search strategy, research questions and the inclusion and exclusion criteria.^16,17^

**Box 2.** The PICO Mnemonic.

Population: Patient with Parkinson’s disease OR atypical Parkinson syndromes, OR their caregivers, OR healthcare workers; Intervention: Any introduction/application/assessment/intervention of palliative care for Parkinson’s disease patients. Context: Care level: all health care settings (place of residence and institutions) and in all levels (primary to tertiary) Outcome: Any reported outcomes, including negative results, linked to timely access to palliative care services.

### Selection criteria

Articles were included if they presented empirical studies about any approach used or implemented to enable better palliative care for people with Parkinson’s disease. Box 3 provides detailed inclusion and exclusion criteria.

**Box 3.** Inclusion and exclusion criteria.

**Inclusion criteria** Patients with Parkinson’s disease OR atypical Parkinson syndromes, OR their caregivers, OR healthcare workers. Any introduction/application/assessment/intervention of palliative care for Parkinson’s disease patients. Palliative care in all health care settings (place of residence and institutions) and in all levels (primary to tertiary) Studies with human participants published in English. *For quantitative designs*, all experimental study designs and observational designs, such as descriptive studies, cohort studies, cross-sectional studies, case studies and case series studies *For qualitative designs*, all studies focussing on qualitative data, such as phenomenology, grounded theory, ethnographic designs or discourse analysis.
**Exclusion criteria** Any other disease population. Palliative care introduction/application/assessment/intervention due to other disease. Any other setting of informal care services (e.g. parish/church group, sport club, etc.) Preclinical studies, conceptual papers, review articles, books, book chapters, book reviews, conference proceedings, editorials, national guidelines and dissertations and all non-peer reviewed publications.

### Study selection

Two independent authors conducted the literature search. The search results were extracted into the review management system Covidence (covidence.org) to screen the titles and abstracts. A total of 62 studies met inclusion criteria. The Covidence systematic review management tool is recommended by Cochrane for conducting systematic reviews.^18^ Conflicts were resolved by a third independent author or through consensus discussions within the consortium (see Supplemental Figure 1: The PRISMA flow diagram).

Quality appraisal was conducted by three independent authors using the standard Quality Assessment Criteria for Evaluating Primary Research Papers from a Variety of Fields (QualSyst).^19^ Consensus discussions were held to solve any interrater disagreements. The Preferred Reporting Items for Systematic Reviews Checklist was used for reporting.

### Evidence synthesis

The data were analysed using thematic synthesis.^20^ Firstly, themes were extracted from literature closely following the body of evidence, and secondly, they were categorised into analytical themes based on group discussions. Finally, the analytical themes were assigned as practice recommendations category of ‘Do’, ‘Do not’ or ‘Do not know’. This allocation was done taking the content, direction and strength of the supporting evidence into account by four independent authors (C.W., M.G., P.P. and S.M.P.) and the PD_Pal consortium representatives (K.R., D.J.P., P.O., Ü.K., A.S., A.A. and S.L.). The latter was formed by clinicians and researchers funded under the European Union’s Horizon 2020 research and innovation programme under grant agreement No. 825785 (www.pdpal.eu). Conflicts were solved through discussion until consensus was reached. A ‘strong’ recommendation was made where there was evidence from a large and consistent body of evidence complemented by expert opinions from the PD_PAL consortium; a ‘moderate’ recommendation was made considering solid empiric evidence from one or more papers plus expert opinions from the PD_PAL consortium; and a ‘tentative’ recommendation where there was limited empiric evidence as perceived by the experts from the PD_PAL consortium (Table 1).

**Table 1.** How categories of recommendation were decided by independent reviewers.

Do	Evidence from a large and consistent body of evidence^a^ on the efficacy of the approach implemented to enable better palliative care for people with Parkinson’s disease complemented by expert opinions from the H2020 PD_PAL consortium
Don’t	Evidence from a large and consistent body of evidence suggesting the lack of efficacy of the approach taken to enable better palliative care for people with Parkinson’s disease complemented by expert opinions from the H2020 PD_PAL consortium
Don’t know	Limited empiric evidence of efficacy of the approach taken and further research is required and/or high-quality published data and research to support the use of this approach complemented by expert opinions from the H2020 PD_PAL consortium
Strength of recommendations	
Strong	Large and consistent body of evidence complemented by expert opinions from the H2020 PD_PAL consortium
Moderate	Solid empiric evidence from one or more articles complemented by expert opinions from the H2020 PD_PAL consortium
Tentative	Limited empiric evidence, but consensus opinion from the authors of this review following critical appraisal of the evidence and complemented by expert opinions from the H2020 PD_PAL consortium

a Here, we follow the format of the systematic practice review already published in this journal. The ‘a large and consistent body of evidence’ refers to the vast majority of literature found in the systematic review (see Supplemental Figure 1: The PRISMA flow diagram).

## Results

A total of 894 articles were retrieved from the search, but only 220 were fully assessed for eligibility after removal of duplicates and irrelevant papers. A total of 157 papers were further excluded as they did not meet the inclusion criteria.

A summary of the recommendations based on the main categories identified through the literature review is presented in Table 2. The strength of each recommendation is also presented in Table 2.

**Table 2.** Summary of recommendations on how to enable better palliative care for people with Parkinson’s disease.

Recommendations	Strength of the evidence
Professional healthcare providers and care coordination	
Do	
1. Provide patients with Parkinson’s disease and their caregivers with clear and timely information on what services or supports are available and how to access them	Strong
2. Provide clear information about the role and duties of the health and social care professionals involved	Strong
3. Palliative care teams for patients with Parkinson’s should be multi-professional and include: physicians, nurses, social care workers, psychologist/psychiatrists, chaplains/spiritual care workers, bereavement support care providers, occupational therapists, speech language therapists, dietitians and care coordinators as part of the multidisciplinary care team	Strong
4. Develop individualised care plans, particularly at the end of life	Strong
5. Promote palliative care awareness and education (e.g., it is not only for patients with cancer; it is not only for patients at the end of life)	Strong
6. Foster access to home-based palliative services, such as hospice care	Moderate
7. Use a care management approach, designating a care manager in charge of palliative care coordination.	Moderate
8. Specialist palliative care services may be beneficial, especially in later stages of the disease	Moderate
Don’t	
1. Provide limited and sporadic contact with healthcare teams (as this resulted in some persons with Parkinson’s disease and Family caregivers felt ‘left alone’ in the face of their illness)	Strong
2. Present different healthcare professionals at every clinic or hospital visits	Strong
3. Show lack of cohesion between services, care continuity, fragmentation of services, time pressure and information discontinuity (as this leads to uncertainty about the support services available and frustration among participants, affecting carers’ ability to care for the person in need of care)	Strong
4. Provide uncoordinated and patchy access to palliative care	Strong
5. Focus the interaction between the specialist, patient and carers on medication, with little or no psychological support or referral to other types of services	Moderate
6. Diffuse responsibilities among healthcare professionals involved in (palliative) care provision	Moderate
Don’t know	
1. Best timing to introduce palliative care into the care provided to patients with Parkinson’s disease	Strong
2. The clear referral cut-off point or triggers for the integration between specialised palliative care and neurology	Strong
3. The role of professional associations and charities in the organisation and provision of (palliative care) in patients with Parkinson’s disease	Tentative
Advance care planning	
Do	
1. Start advance care planning discussion before the cognitive decline begins	Strong
2. Start advance care planning discussion when the caregiver (and the patient) is ready for it	Strong
3. Involve Parkinson’s disease specific aspects in advance care planning	Moderate
4. Use motor and non-motor symptoms as triggers for advance care planning	Moderate
Don’t	
1. Force advance care planning conversations on people	Strong
2. Avoid difficult conversations	Strong
Don’t know	
1. When and how many people want to have the advance care planning talk	Tentative
2. How death related traumata affects the willingness to have the advance care planning	Tentative
3. Which healthcare provider has the authority to have the advance care planning talk	Tentative
4. If advance care planning should or could be conducted by trained volunteers	Tentative
Involvement of and care for informal caregivers	
Do	
1. Recognise that caregivers have a crucial role to the optimal patient care	Strong
2. Acknowledge that Family carers need support from both social care and the health system	Strong
3. Assess caregiver experiences and support needs regularly	Strong
4. Include caregivers into care-related discussions	Strong
5. Initiate conversations about end-of-life care	Strong
6. Offer psychological support or referral to other services	Strong
7. Address anticipatory grief and consider bereavement care	Strong
8. Suggest regular breaks from caring and offer respite care services	Moderate
Don’t	
1. Avoid conversations about end-of-life wishes	Strong
2. Abandon the caregiver if patients want to stay at home despite their deteriorating health	Strong
3. Focus only on medication	Moderate
Don’t know	
1. Caregivers motivations to become caregivers	Tentative
2. How inconsistent support from the fragmented health system affects caregivers physically, emotionally, socially, economically, spiritually	Tentative
3. If participation in the palliative care model is reducing the burden on caregivers	Tentative
4. How people who do not have caregivers cope	Tentative
Triggers for palliative care referral	
Do	
1. Investigate the impact of the diagnosis on patient and caregivers	Strong
2. Involve since the diagnosis a multidisciplinary team to target patient’s needs	Strong
3. As condition and its burden progress, consider the introduction of palliative care	Strong
4. Focus on the quality of life of the patient and his or her family	Strong
5.Be sensitive for key crisis times for extra support and introduce palliative care	Strong
6. If patient presents: Hoehn & Yahr ⩾ 3, accompanied by non-motor symptoms non-controllable with pharmacological therapy (dysautonomia, neuropsychiatric symptoms, dysphagia, pain and weight loss) consider palliative care referral	Strong
7. Use specific scales to assess patient’s needs	Moderate
8. Encourage patient and his family members to start discussions about preferences and goals of care at early stages of the disease	Moderate
9. Provide a network of services on the territory: PD patients, especially if homebound, need integrated care	Moderate
Don’t	
1. Avoid realistic conversation about disease stage, prognosis, possible therapeutic options and disadvantages of proposed treatment	Strong
2. Avoid addressing end of life with patients and caregivers, they need to be prepared	Strong
3. Delegate responsibilities for the introduction of palliative care	Strong
4. Wait for cognitive decline to arise before discussing care goals and advanced directives	Strong
5. Forget that the goal is the maximisation of quality of life	Strong
6. Provide regular surveillance to patient’s needs	Moderate
Don’t know	
1. It is appropriate to involve a palliative care approach since the diagnosis	Tentative
2. When is needed the support of specialist palliative care	Tentative

### Professional healthcare providers and care coordination

Persons with Parkinson Disease and their carers have complex needs that require a person-centred, well-integrated and coordinated, multi-sectoral and interdisciplinary approach. The articles included in the analysis suggest that there is a need for clear and timely information on available health and social care resources, as well as on the roles and duties of different stakeholders involved in the care process.^21–25^ It is paramount to ensure that these patients and their families have access to home-based palliative care services, such as hospice care.^22^ According to included articles, these palliative care teams need to be multi-professional, including a palliative care physician, nurse, social care worker, chaplain/spiritual care worker, bereavement support care providers, occupational therapists, speech language therapists and dietitians.

A care management approach should be embraced, designating a care manager in charge of palliative care coordination.^23^ Specialist palliative care provision may be beneficial, particularly at the later stages in the disease trajectory including end-of-life care.^26^

The integration of palliative care into the care provided to patients with Parkinson’s disease and their families encompasses many challenges and uncertainties. In fact, according to the existing literature the best timing to introduce palliative care is yet unidentified, and no consensus on clear cut-off points or triggers for palliative care referral have been identified in the existing literature.^9,21–23,27–33^ Evidence on the role of professional associations and charities/non-governmental institutions in the organisation and provision of palliative care for people with Parkinson’s disease is also required.^32^

### Advance care planning

There is disagreement about the right time to bring in advance care planning. One way to make advance care planning in a non-disease specific manner even more acceptable would be to start this process while the patient is still healthy.^34^ Advance care planning discussions should be made with the neurologist or primary care physician,^34^ and supplemented with disease-specific aspects.^27,35^ Involving the family in the discussion is desired by many patients,^36^ but based on the experience of experts from the PD_PAL consortium, other patients do not want to burden their close ones. The patient must be ready for it, which can depend on age, personality and stage of disease: patients can be more prone to discuss those aspects when the illness course has severely affected their lives.^21,24,36^ Patients and relatives often shy away from end-of-life discussions and prefer to adopt a ‘living in the moment’ attitude.^24,36^

In case cognitive impairment occurs or apathy is present as a symptom of the disease, this can deprive the patient of the ability to make decisions for themselves so early involvement of advance care planning is recommended by some authors.^37,38^ A list of symptoms and problems, which may appear alongside hallmark PD symptoms, could serve as potential indicators for initiating advance care planning discussions. These symptoms include hopelessness or fear of the future, frequent falls (e.g. resulting in hip fractures), dysphagia or pneumonia, cognitive deficits/neuropsychiatric problems and unplanned hospitalisation. At least two of these symptoms should be present to warrant consideration.^11,23^

### Involvement of and care for informal caregivers

In the palliative care model, regular communication with patients with Parkinson’s disease and their caregivers throughout the illness is key. As important and central as caregivers are to the optimal care of the patient, they also need support and care. Limited and sporadic contact with healthcare teams left some patients with Parkinson’s disease and their caregivers feeling ‘left alone’.^21,24^ Moreover, there is a lack of continuity of care and information breakdowns.^23^ Patients and caregivers felt unsupported when they had to deal with different healthcare professionals, each time they visited a clinic or hospital. The lack of cohesion between services leads to uncertainty about the support services available and resulted in frustration for participants and affected caregivers’ ability to care for the person in need of care.^21,24^ The dispersion of responsibilities between the health professionals involved leads to uncoordinated and patchy access to palliative and clinical care.^21–23^ Caregivers should be included in discussions with the patient’s permission, especially as they often want to make plans for the later stages of care earlier than the patients.

Another impeding factor to accessing appropriate palliative care is the fact that conversations about end-of-life care are extremely uncomfortable for many people affected and are therefore often postponed. One study explored the reasons for postponing conversations about end-of-life wishes. The reasons given were that the patient could not cope, that patients should not be deprived of hope, that the patient is not suffering and also that these conversations take a lot of time.^29^ If the interaction is very brief or focusses only on medication, with little or no psychological support or referral to other services, there is little opportunity to plan care appropriately. This can lead to *ad hoc* use of services.^22^ Variations in treatment guidelines among patients, relatives and health professionals can lead to disagreements about when to transition from standard curative therapy to palliative care.^29^ Many people with Parkinson’s disease prefer to remain at home as their health declines, but the lack of home care services can hinder access to palliative care.^22^ Palliative care extends beyond the patient’s death, and as such, referring caregivers to bereavement counselling services can be considered a facilitating aspect for palliative care provision. Addressing anticipatory grief and ensuring the availability of bereavement counselling before the patient’s death is fundamental as well as ongoing support after their passing.^22^ One study also highlights the challenges faced by individuals without formal caregivers, making it more challenging for them to access palliative care. In such cases, the responsibility for organising and facilitating this support often falls to caregivers.^21^

### Triggers for palliative care referral

A growing body of research indicates that people with Parkinson disease require a palliative approach to their treatment, but there is a great of heterogeneity in the attempt to define indicators for referral, though it has been proposed that it should begin when the diagnosis is established.^21,28,33,39^ Several tools have been identified as useful and practical for assessing the need for referral to palliative care in the care of patients with advanced Parkinson’s disease: the use of scales such as the Edmonton Symptom Assessment System adapted for PD (ESAS-PD), the Palliative care Outcome Scale (POS) and the Palliative Care Needs Assessment Tool (PC-NAT, modified for PD), can help identify symptoms not elicited in routine care.^9,11,40,41^

The palliative care intervention phase has generally been ascribed as belonging to advanced or late-stage Parkinson’s disease, defined as with functional disability expressed by the Hoehn and Yahr (H&Y) scale between III and V,^9,38,42^ increased progressed non-motor symptoms, Parkinson’s disease related complications, such as dysphagia, weight loss, recurrent infections, dysphagia, neuropsychiatric problems and ineffectiveness of pharmacological therapy. The presence of cognitive, behavioural disorders and an increased need for help with activities of daily living have been described as a warning sign to initiate a palliative approach and discuss the advance care planning to ensure that the patient can express his or her wishes while still able to attain them.^29^

Disease progression, as expressed by increased financial, emotional and social burden on caregivers, has been cited as a key issue in several studies,^30,43,44^ highlighting the need for more structured professional care when informal caregivers cannot continue to fulfil their role and patients' needs exceed the care that can be provided at home.

Increased use of professional services and transition of care needs expressed by heightened use of health care facilities have been reported as a criterion for referral to palliative care.^43–45^ Specifically, these studies have described increased strain when leaving home, requiring assistive devices, assisted transportation and the need for additional professional help, as well as increased use of health and emergency services, as expressed by recurrent hospitalisations and admission to nursing homes.

A general progression towards a different care goal, understood as maximising comfort, has also been ascribed as a key point for referral.^45^

The involvement of specialist palliative care, in addition to the generalist palliative care approach, has been proposed in cases of advanced Parkinson’s disease complicated by severe conditions, such as aspiration pneumonia, swallowing difficulties and ethical dilemmas or complex discharges.^21^

## Discussion

This systematically constructed practice review provides healthcare professionals with a pragmatic overview of the approaches to enable better palliative care for people with Parkinson’s disease. These are systematised into the ‘Do’, ‘Do not’ or ‘Do not know’ on four core dimensions: Healthcare providers and care coordination; Advance Care Planning; Involvement of and care for informal caregivers; and triggers for palliative care referral. Recognition of Parkinson’s disease as a neurodegenerative disease that benefits from the palliative care approach is paramount together with the need to implement person-centred care approaches, such as Advance Care Planning.

### Recognition of Parkinson’s disease as neurodegenerative disease that benefits from the palliative care approach

In 2006, WHO acknowledged that the burden of neurological disease is substantially underestimated by traditional epidemiological and health statistical methods that consider only mortality rates, not disability rates.^15^ Clinicians argue that a clearer definition of palliative care is needed, as it is still perceived as a model of care applied in the last weeks of life. Modern palliative care can be offered alongside treatments that target the underlying disease and, depending on the complexity of the pre-diagnostic phase in people with Parkinson’s disease, it can be useful from the time of diagnosis.

The International Association for Hospice and Palliative Care (IAHPC) consensus definition states that (1) palliative care is applicable in all healthcare settings (residential and institutional) and at all levels (primary to tertiary); (2)palliative care can be provided by professionals with basic training in palliative care and (3) specialised palliative care with a multi-professional team is required for referral of complex cases.^46^ Parkinson’s disease is a chronic neurodegenerative disease that benefits from the palliative care approach. Throughout the course of the disease, the palliative care model should be applied, and palliative care specialists should be consulted and involved in care coordination as needed.

The patient’s problems should be considered from the patient’s perspective in order to apply the concept and practice of person-centred care. Palliative care should be continuous and provided throughout the disease trajectory, depending on patients’ needs. In particular, the value of the palliative approach increases gradually as patients move into the late stages of the disease, as the needs grow exponentially. The conservative view of palliative care as end-of-life care is outdated.^47^ One should try to emphasise the systemic manifestation of the disease from the beginning (or even before), with the need for multi-professional and interdisciplinary care, including advance care planning when the symptom burden becomes increasingly difficult to control.

The identified lack of palliative care education among all stakeholders involved in the care of people with Parkinson’s disease^8^ and their caregivers were addressed by the PD_Pal consortium with an evidence-based curriculum toolkit ‘Best Care for People with Late-Stage Parkinson's Disease’. The toolkit is based on recommendations and guidelines for the training of clinicians and other health professionals involved in palliative care, on teaching materials developed for patients and caregivers in recent research projects and on consensus meetings of leading experts in the field.^48^ Completion of this course will enhance clinicians' understanding of (1) the philosophy of palliative care, (2) increase their skills in symptom assessment and management, (3) promote the creation of care plans based on the wishes of patients and families, incorporating multi-professional and interdisciplinary approaches and (4) teach why it is necessary to be able to listen and self-reflect.^49^

### Advocating for advance care planning

In reality, advance care planning is commonly perceived as ‘nice to have’. Although in many countries there is no place or space for such discussions, it nevertheless has little or no meaning when patients are referred to different professionals who do not work together to provide multidisciplinary care. Timely and well-executed advance care planning can empower patients and caregivers, allowing them to maintain a sense of control over their lives. It also enhances the therapeutic alliance with healthcare professionals, making patients feel heard and respected as well as ensuring that individuals’ wishes are respected.^36,50^ All these aspects can contribute to a more peaceful and less fearful approach to end of life.^51^

Access to training and education in palliative care, including advance care planning, is limited.^52^ Therefore, physicians need specific guidance on when and how to start these conversations with patients in neurology. It remains a challenge, especially for less experienced physicians, to balance timely conversations with hope.

This practice review shows that advance care planning should define how to optimise care when people suffer from their illness. This is usually not a traditional palliative approach, but rather other types of advanced disease management, focussing on non-motor symptoms and quality of life. Advance care planning should include also Parkinson’s-specific aspects, for example, levodopa carbidopa intestinal gel (LCIG), deep brain stimulation (DBS), swallowing disorder, changes in personality and neuropsychological symptoms and bladder and rectal problems.^27^

In fact, this practice report shows that it is difficult to determine when to start advance care planning. However, it is important to let the caregiver and the patient decide (in that order) when this conversation should begin. Under no circumstances should this conversation be forced on them. Clinicians and researchers should not underestimate the burden that advance care planning can place on some patients when talking about end-of-life care, as many of them feel that this type of service is not necessary for terminally ill patients.

It should also be emphasised that there are significant differences in terms of the implementation of advance care planning and attitude towards it across health systems. According to this practice review, advance care planning should ideally commence when at least two of the following symptoms or problems occur: (a) hopelessness or fear of the future, (b) frequent falls, potentially linked to hip fractures, (c) dysphagia or pneumonia, (d) cognitive deficits and/or neuropsychiatric issues and (e) unplanned hospitalisations.^45^

### Family caregivers are the care coordinators

Caregivers play a central role as partners in the palliative care model. At the same time, they require ongoing support throughout the course of the illness.^30,39^ It is well known that the increasingly complex care needs of the aging population, coupled with the expected decline in the availability of informal carers, will create a ‘care gap’, posing significant challenges to the future sustainability of global health and social care systems.^53^ Policies underpinning support for caregivers should prioritise the perception of care as a public responsibility rather than solely a family one. Pressure on family members by formal support providers and policies often assumes that individuals are willing to provide unpaid informal care, even when they may not want to do so.^54^ Caregivers need support from both social and health care systems. The need for care provided by caregivers increases as the disease progresses, especially in the late stages of Parkinson’s disease. Participation in the multi-professional and interdisciplinary palliative care model is intended to reduce the burden on caregivers. Yet caregivers often experience inconsistent and fragmented support from the health care system.^39^

The financial costs borne by caregivers and their care recipients need to be taken seriously.^54^ The European Commission has stressed the importance of supporting caregivers in national policies to maintain their motivation to care.^55^ Personal values, but also social and cultural norms, sometimes force people to become informal caregivers. Therefore, caregivers’ experiences, resulting needs and support planning need to be assessed regularly. Policies that target carers’ beliefs and expectations, coping skills or social resources can help maintain motivation and readiness to care. Any policy that supports carers should also include the provision of breaks from caring, such as breaks from caring and respite services.^54^

### What this practice review adds?

To the best of our knowledge this is the first practice review that systematises the existing evidence about the approaches to provide better palliative care for people with Parkinson’s disease, providing pragmatic recommendations. Compared to other reviews focussed on the integration of palliative care in Parkinson’s disease, this practice review systematised the main findings around four core dimensions providing a comprehensive list of ‘Do’, ‘Do not’ or ‘Do not know’ for each dimension: Healthcare providers and care coordination; Advance Care Planning; Involvement of and care for informal caregivers; and Triggers for palliative care referral.

Nevertheless, this practice review is not without limitations. On the one hand, the existing evidence about this topic is heterogenous and included articles had different quality levels. On the other hand, the strength of evidence relies on the expertise (clinical, scientific and academic) of the members of the PD_PAL consortium.

## Conclusions and implications for future practice

Recognising Parkinson’s disease as a neurodegenerative condition that benefits from a palliative care approach throughout its entire trajectory is of paramount importance, along with the imperative need to implement person-centred care approaches. This practice review has provided a comprehensive overview and presented pragmatic recommendations across four core dimensions deemed to be crucial for enhancing palliative care for people with Parkinson’s disease: healthcare providers and care coordination, advance care planning, involvement of and support for informal caregivers and triggers for referral. To improve the care experience for people with Parkinson’s disease, it is essential to provide clear and timely information about available services and the roles of healthcare professionals involved. Multi-professional palliative care teams should be established, individualised care plans developed and awareness and education on palliative care promoted. Patients’ goals and values should be respected and included in effective advance care planning, which should be initiated before cognitive decline, respecting the readiness of patients and caregivers and incorporating Parkinson’s disease-specific aspects. When considering palliative care referral for people with Parkinson’s disease, assessing the impact of the diagnosis, involving multidisciplinary teams from the early stages and focussing on the patient’s and family’s quality of life are essential. Key crisis times should be identified for additional support and specific criteria can guide referral to palliative care. Furthermore, regular assessments of caregivers’ experiences and needs along disease course are essential to offer support and monitor their health.

In addition to the recommendations outlined above, further research is warranted to develop specific interventions aimed at enhancing symptom management, particularly towards the end-of-life phase for people with Parkinson’s disease. Additionally, strategies for fostering the seamless integration of palliative care into the care continuum for these patients and their families require further investigation. These efforts collectively aim to provide comprehensive, compassionate and patient-centred care for people with Parkinson’s disease and their caregivers.

## Supplemental Material

sj-pdf-1-pmj-10.1177_02692163231214408 – Supplemental material for A systematic practice review: Providing palliative care for people with Parkinson’s disease and their caregivers Click here for additional data file. Supplemental material, sj-pdf-1-pmj-10.1177_02692163231214408 for A systematic practice review: Providing palliative care for people with Parkinson’s disease and their caregivers by Michela Garon, Christiane Weck, Kristina Rosqvist, Per Odin, Anette Schrag, Ülle Krikmann, David J Pedrosa, Angelo Antonini, Stefan Lorenzl, Sandra Martins Pereira and Piret Paal in Palliative Medicine
