# Supplementary figures and images for: A systematic practice review: Providing palliative care for people with Parkinson’s disease and their caregivers
Source: Palliat Med. 2023 Dec 6;38(1):57–68. doi: 10.1177/02692163231214408 (PMC10798024; doi:10.1177/02692163231214408)

Supplementary figure 1. PRISMA Flow Diagram

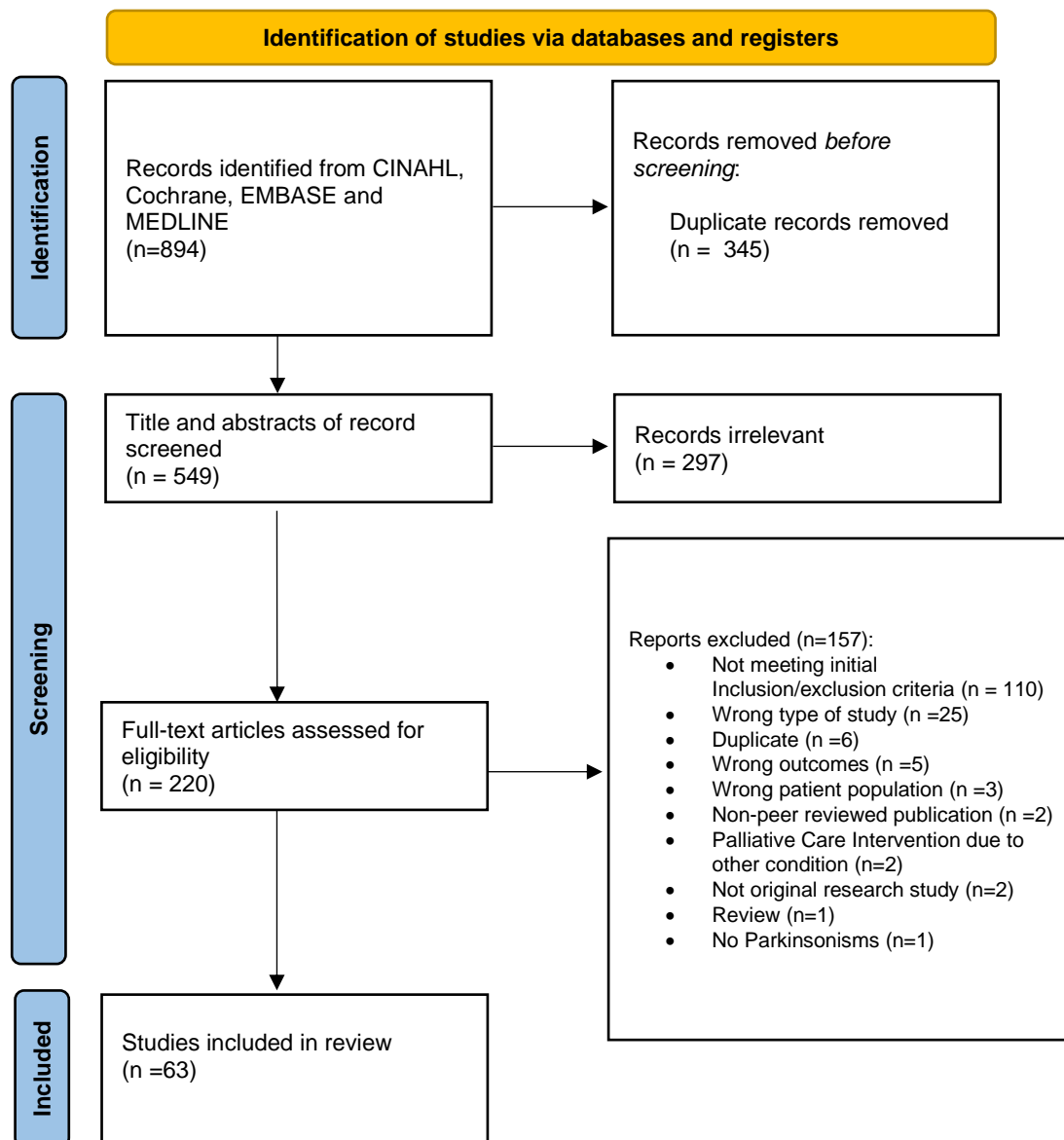

Supplement: sj-pdf-1-pmj-10.1177_02692163231214408 – Supplemental material for A systematic practice review: Providing palliative care for people with Parkinson’s disease and their caregivers [file sj-pdf-1-pmj-10.1177_02692163231214408.pdf]
